# Factors Affecting Placement of a Child with Intellectual Disability

**DOI:** 10.1100/tsw.2005.48

**Published:** 2005-05-06

**Authors:** Isack Kandel, Joav Merrick

**Affiliations:** ^1^ Faculty of Social Science, Department of Behavioral Sciences, Faculty of Social Science, Academic College of Judea and Samaria, Ariel, Israel; ^2^ National Institute of Child Health and Human Development and Center for Multidisciplinary Research in Aging, Faculty of Health Sciences, Ben Gurion University of the Negev, Beer-Sheva, Office of the Medical Director, Division for Mental Retardation, Ministry of Social Affairs, Jerusalem, Israel

**Keywords:** human development, public health, disability, intellectual disability, mental retardation, Israel

## Abstract

Parents of disabled children often face the question whether or not to keep the child at home or to place them. The choice between the two alternatives resides with the parents and various factors influence their decision. Several researchers have identified these factors, which include child-related parameters, family and parental attitudes, the influence of the social environment, and the external assistance provided to the family. In a pilot study, we attempted to isolate the main factors involved in the parental decision either to keep the child at home or place the child by examining a sample comprised of 50 parents of children suffering severe intellectual disability studying in a special education school and 48 parents of adults with intellectual disability working in sheltered workshops. Each parent filled out a questionnaire used in a study in the United States and results of the research indicated parental-related factors as the dominant factors that delayed the placement of their child in residential care; guilt feelings were the main factor.

## INTRODUCTION

The birth of a child with a disability is a traumatic event. The family often begins with a sense of quasi-mourning for the child who was not born healthy and also experiences the daily stress caused by raising an exceptional child at home. The temptation to alleviate the stress by placing the child in an institution begins at this stage. The family's ability to gear up and adjust to this is contingent on resources available to the family, its inner strength, and the ability to mobilize support both internally and externally. On the birth and realization that the child was born with a disability, parental expectations in regard to the future must be curbed; they must relinquish normal expectations (happiness, satisfaction, self-realization) and instead prepare for social isolation, impaired intimate relations, and effects on healthy siblings, which find expression in school and society[1]. In families with exceptional children, the feeling of coherence is impaired and events in their world are grasped with diminished clarity[2].

Cole and Meyer[3] maintain that an examination of preference between raising the child in an institution or at home reveals that the decision will be predicated on the degree of the family's contribution. A family laden with previous pressures and problems will cope by removing the added source of tension. Families enjoying high internal resources display greater determination to keep the child at home.

Parental reactions can be classified into several categories on the birth of a child with a disability: they react constructively, they become alienated, or they exhibit denial. Wolfensberger and Kurtz[1] suggested three forms of crisis that the family undergoes: novelty crisis, personal crisis, and reality crisis. The emotional reactions exhibited by parents on learning that their child is intellectually disabled include shock, denial, depression, guilt, anger, grief, and anxiety[4]. Farber[5] encountered emotional reactions that typified a state of bereavement. Parents, mainly the mothers, reported chronic fatigue[2], but the character and the intensity of these reactions can be affected by different variables such as age, gender, social, and religious background. In professional literature, an attempt has been made to characterize these variables and the means of coping with them in the course of making the decision between raising the child at home or in an institutional framework.

## FAMILY AND SOCIAL ASPECTS OF DISABILITY

Since disability usually is a chronic condition, the family must prepare for a long-term burden on certain realms of its life. A means of coping with this burden could provide the best reason in the decision process on whether or not to place the child ("the source of the burden") in an institution or residential care center. Stress, fatigue, and tension are high, especially among the mothers of children with disabilities resulting from the daily work routine surrounding the exceptional children. Execution of regular daily tasks becomes impossible in cases where the degree of the child's disability is severe or in the case of added behavioral problems[6,7,8,9].

Beckman[6] found a correlation between the outward expressions of disability and the measure of family tension. A correlation was indeed found between characteristics that imposed a burdensome day-to-day care regimen on the family and, as a result of this burden, a tendency to place the child in an institutional framework. A recent study of 100 mothers with adult children with intellectual disability[7], a comparison between mothers who had placed their adult child in a community arrangement and those who had kept their child at home, showed no significant difference for most indicators, but mental health, stress, hardiness, and social support were highly intercorrelated.

It is obvious that the effect on siblings is much stronger if the child remains within the family framework and the siblings are with him or her all the time. Parents, contemplating the hard dilemma of whether or not to place the child out of the home, take the issue of the effect on siblings very seriously in their decision[10]. Several researchers have examined the effect of the disability on siblings and two currents could be found: the negative flow or the damaging influence, pernicious to the siblings, as opposed to a positive flow, which emphasizes how the disabled child can contribute to the development of the siblings[10]. The daily burden involved in caring for the disabled child affects demands made on the siblings to become practical partners in the division of household tasks. Thus, the sibling is "pushed" into a premature parental role, which sometimes is a burden of responsibility for which he or she does not possess the ability or strength[10]. The closer the disabled child is in age to the healthy sibling, the greater the strength required by the latter to adjust and become accustomed to the role. In summary, we can observe that these variables exert a practical effect on the parental decision regarding the arrangement and placement and future way of life.

The birth of a child with a disability can also affect the parental married life[10] in several ways: it evokes strong feelings on both sides, acts as a debilitating symbol of mutual failure, reshapes the arrangement of family life, and creates a fertile ground for conflict. Families with sound marital relations display much more strength in coping with the disabled child at home rather than sending him or her to an institution. Graliker et al.[11] found that 68% of the families who decided to send their child to an institution had family background problems, some of which pre-existed the birth of the child.

Aside from the nuclear family, there are relations, grandparents, uncles, friends, and neighbors who influence how the family deals with their child's disability and many families find support within the extended family[2]. It has been established that the tenor of relations with the rest of the family is significant. It appears that the maternal grandmother has a supportive and auxiliary role within the family coping with disability. She frequently encourages raising the child at home rather than sending him or her to an institution. People in the near surroundings of the family can also help; parents of disabled children often observe how others regard their child and behave towards him or her naturally and without prejudice. They inquire into the welfare of the parents without displaying feelings of pity. All this facilitates easy and, if necessary, supportive relations, which in turn alleviates the parents' dilemma to keep the child at home[10].

One of the main issues on which professionals have a decisive influence regards the future of the disabled child. If family resources are larger and are utilized, the family has a better chance to cope with the situation. Families who lack these resources and are unfamiliar with the ways in which they can utilize community services continue to feel pressured and prepare to place their children in institutions. Usually, the first contact is with medical services with regard to the primary diagnosis and the role of the community services is to act as a support system shaping the possibility of coping. The basic assumption is that early intervention and support in the initial stages can help parents cope with their problems. Parental expectations are met when they receive a clear explanation of the child's problems, are treated with respect, and get detailed information regarding the care regimen and tangible help[10].

## HOME VERSUS RESIDENTIAL CARE

Over time, the approach has varied in regard to raising the disabled child at home or in a residential care center. The prevalent care policy today recommends leaving the disabled child at home as long as possible. This assumption is grounded on the principle of normalization and on the assumption that the home is the best place for the disabled[10,11,12]. The trend of normalization[1], originating in Denmark in the 1960s, posits that leaving those with disability in their home is preferable to placing them in an institution. All individuals, irrespective of the severity of their disability, should be able to fulfill the potential they have within in the community they live in, but on the other hand, this means that society has to be able to support and provide the resources for the families, which is not always possible in every country.

As mentioned above, one can focus on three main components that influence the decision-making process with regard to raising the disabled child in the family home: the disabled child's characteristics, family characteristics, and factors external to the family. Tausig[13] found that among younger disabled individuals (under 21 years), behavior problems were most important for the decision of placement and for older individuals (over 21), disruption of family relations and perceived burden of care were more important. Others[10] found that out-of-home placements were associated with male gender, challenging behavior, and the absence of one or both parents.

## A PILOT STUDY

In order to examine the reasons for parental decisions to leave the disabled child at home or to place them, we assumed that the reasons influencing the decision to leave the child at home were: (1) sentiments for the child, (2) the family constellation, (3) the behavior of the child, and (4) external assistance for the family. We also assumed that there would be a gender preference for girls and an age component.

The study population comprised two groups: 50 parents of children with severe intellectual disability learning in special education schools (group 1) and 48 parents of adults with intellectual disability (mild to moderate) over the age of 18 working in sheltered workshops (group 2). In order to examine the assumptions of the research, we used a questionnaire adapted to this population (see Table 1)[12] with 20 structured questions and 1 open-ended question according to a Likert scale.

**TABLE 1**
**Questionnaire to Determine Factors Affecting the Decision to Keep a Child with Disability at Home or to Choose Placement[12]**

| Gender of child___________<br>Age of child _____________ | Very Intense Influence 1 | Intense Influence 2 | Average Influence 3 | Weak Influence 4 | No Influence 5 |
|---|---|---|---|---|---|
| 1. I am very attached to my child | | | | | |
| 2. Thoughts of someone else to care for my child | | | | | |
| 3. My son/daughter will not understand if he/she is placed in an institution | | | | | |
| 4. There is a framework suitable for my son/daughter (e.g., school) | | | | | |
| 5. Thoughts of placing my son/daughter in an institution makes me feel guilty or that I am not a good parent | | | | | |
| 6. My other children get on very well with their disabled sibling | | | | | |
| 7. I realized that I could not see my son/daughter often | | | | | |
| 8. My partner helps a great deal with my son/daughter at home | | | | | |
| 9. My son/daughter learns skills that make it easier for him/her to be at home | | | | | |
| 10. The welfare services enable me to rest from constant care at home | | | | | |
| 11. I have a babysitter or caregiver | | | | | |
| 12. My son/daughter displays severe behavior problems | | | | | |
| 13. I could not find a suitable institution | | | | | |
| 14. Raising my son/daughter at home is no different from raising my other children | | | | | |
| 15. We have not found a suitable institution for my son/daughter | | | | | |
| 16. My partner’s attitude towards the idea of placing our son/daughter in an institution | | | | | |
| 17. A friend advised raising my son/daughter at home | | | | | |
| 18. Professionals advised me to leave my son/daughter at home | | | | | |
| 19. I do not know how to find a place in an institution | | | | | |
| 20. My religious beliefs will make it hard in an institution for my son/daughter | | | | | |
| 21. Miscellaneous ---------------- | | | | | |

## RESULTS AND DISCUSSION

Both populations examined with this questionnaire[12] noted nearly the same reasons for keeping their disabled child at home and we found the following factors prevented them from sending the child to a residential care center according to the following ranking: feelings of guilt and sentiments for their child, family attitude, and external assistance or support (see Table 2). We found correlation with the results found by Bromley and Blacher[12], but were interested to see that external support was less important than feelings of guilt, family attitude, and the functional level of the child. This could indicate that support services in Israel are inferior to their counterparts in the United States, that the parents in Israel trust them less, or that the family support system is stronger in Israel.

**TABLE 2**
**Reasons the Parents Gave for Their Decision to Keep Their Disabled Child at Home According to the Questionnaire Given[12]; Scores on a Likert Scale of 1–5 (1 = Strong Influence and 5 = No Influence)**

| Results / Population | General Average | Feelings of Guilt and Sentiment for the Child | Family Attitude | Functional and Behavioral Level of the Child | External Assistance Services for the Family |
|---|---|---|---|---|---|
| Group 1 | 3.0 | 2.1 | 2.8 | 3.3 | 3.8 |
| Group 2 | 3.2 | 2.3 | 2.9 | 3.6 | 4.0 |

The breakdown of answers provided by the parents on the basis of the age variable of the disabled child did not display any remarkable difference between the two groups. The average results for parents with either a disabled child (group 1) or a disabled adult (group 2) were equivalent with a small difference of 0.2 and similar to other studies[13].

Differences were found between parents of girls and parents of boys in relation to the decision to leave them in the home environment. The tendency to leave the girl at home was higher (average 3.3 with standard deviation of 1.3). A correlation was found the study by Frey et al.[14], who encountered a difference between pressures and distress felt by parents of girls as opposed to parents of boys with disability. This can be attributed *inter alia* to higher expectations with respect to boys, who may be able to maintain themselves in the future, whereas with girls, feelings tend towards protectiveness and pity.

A recent study of help by siblings of children with disability[15] showed that siblings of brothers and sisters with disability engaged in higher levels of perceived emotional support and custodial care, but not information giving or tangible aid. In regard to sibling variables, age was not a predictor of providing emotional support, nor did it predict custodial care. Girls were more apt to help in accordance with other research, where girls and women showed higher levels of empathy and helping than boys and men.

## CONCLUSIONS

We wanted to look at the main factors dissuading parents from placing their disabled child in residential care and instead preferring to keep the child in the home environment. In order to find data on this issue, we conducted a pilot study with 50 parents of children with severe intellectual disability learning in special education schools (group 1) and 48 parents of adults over the age of 18 with intellectual disability (mild to moderate) working in sheltered workshops (group 2) using a questionnaire used in a study from the United States[12].

Several assumptions were investigated, but the principal assumption was that one could rank the factors according to their intensity in explaining the parents' considerations regarding institutionalization for their child. The findings revealed that the most dominant factor in a decision to leave the child at home was the emotional factor: the relationship to the child and feelings of guilt. The family factor placed second and the behavioral factor of the disabled child was ranked third. The factor occupying last place was the measure of external assistance and support that the family received.

## BIOSKETCHES

**Isack Kandel**, **MA, PhD,** is senior lecturer at the Faculty of Social Sciences, Department of Behavioral Sciences, the Academic College of Judea and Samaria, Ariel. During the period 1985–93, he served as the director of the Division for Mental Retardation, Ministry of Social Affairs, Jerusalem, Israel. E-mail: Kandeli@aquanet.co.il

**Joav Merrick, MD, DMSc,** is professor of child health and human development affiliated with the Center for Multidisciplinary Research in Aging, Zusman Child Development Center, Division of Pediatrics and Community Health at the Ben Gurion University, Beer-Sheva, Israel; the medical director of the Division for Mental Retardation, Ministry of Social Affairs, Jerusalem; and founder and director of the National Institute of Child Health and Human Development, Faculty of Health Sciences, Ben Gurion University of the Negev. He has numerous publications in the field of child health and human development, rehabilitation, intellectual disability, disability, health, welfare, abuse, advocacy, quality of life and prevention. Dr. Merrick received the Peter Sabroe Child Award for outstanding work on behalf of Danish Children in 1985 and the International LEGO-Prize ("The Children's Nobel Prize") for an extraordinary contribution towards improvement in child welfare and well being in 1987. E-mail: jmerrick@internet-zahav.net. Website: www.nichd-israel.com
